# Fine mapping a quantitative trait locus, *qSER-7*, that controls stigma exsertion rate in rice (*Oryza sativa* L.)

**DOI:** 10.1186/s12284-019-0304-z

**Published:** 2019-07-09

**Authors:** Yi Liu, Anning Zhang, Feiming Wang, Deyan Kong, Mingshou Li, Junguo Bi, Fenyun Zhang, Jiahong Wang, Xingxing Luo, Zhongquan Pan, Xinqiao Yu, Guolan Liu, Lijun Luo

**Affiliations:** 1 Huazhong agricultural university, Wuhan, 430070 People’s Republic of China; 2 Shanghai agrobiological gene center, Shanghai, 201106 People’s Republic of China

**Keywords:** Rice (*Oryza sativa* L.), Quantitative trait locus, Stigma exsertion rate, Fine mapping

## Abstract

**Background:**

Stigma exsertion rate (SER) is a key determinant of outcrossing in hybrid rice seed production. A quantitative trait locus (QTL) for stigma exsertion rate in rice, *qSER-7*, has previously been detected on chromosome 7 by using a F_2_ population derived from two indica cytoplasmic male sterility (CMS) maintainers, Huhan 1B and II-32B.

**Results:**

The chromosomal location of *qSER-7* was precisely delimited by fine-scale mapping. Near-isogenic lines (NILs) were established, one of which isolated the locus in the *qSER-7*^II-32B^ line, which contains an introgressed segment of II-32B in the Huhan 1B genetic background, and exhibits a significantly higher stigma exsertion rate than that of the recurrent parent. Using 3192 individuals from the BC_4_F_2_ segregation population, the QTL *qSER-7* was narrowed down to a 28.4-kb region between the markers RM3859 and Indel4373 on chromosome 7. According to the rice genome annotation database, three genes were predicted within the target region. Real-time PCR analysis showed significantly higher expression levels of *LOC_Os07g15370* and *LOC_Os07g15390* in II-32B than in Huhan 1B. *LOC_Os07g15370(OsNRAMP5)* was a previously reported gene for Mn and Cd transporter. The stigma exertion rates of *OsNRAMP5*-overexpressing plants were significantly higher than that of wild type plants, in contrast, a T-DNA insertion mutant *osnramp5* showed a lower stigma exertion rate.

**Conclusions:**

In the present study, the QTL *qSER-7* was isolated to a region between the markers RM3859 and Indel4373. Two candidate genes were selected based on the expression difference between the two parents, which can facilitate the further cloning of the gene underlying the quantitative trait associated with *qSER-7* as well as the marker-assisted transfer of desirable genes for stigma exsertion rate improvement in rice.

**Electronic supplementary material:**

The online version of this article (10.1186/s12284-019-0304-z) contains supplementary material, which is available to authorized users.

## Background

Rice (*Oryza sativa* L.) is a major cereal crop that feeds most of the world’s population. By 2030, rice production must increase at least 40% in order to satisfy demands of the ever-growing human population (Khush 2005). Accordingly, it is inevitable that rice production will have a direct effect on global food security and social stability. The commercialization of hybrid rice initiated in China in the 1970s has greatly contributed to the increase in rice yield (Stuber 1994; Yuan 2004). The basis of utilizing heterosis in rice is to use male sterile lines (cytoplasmic male sterility lines or thermo-sensitive genic male sterile lines) as the female parents. However, despite continuous improvements in cultivation techniques for F_1_ seed production over the last 10 years, the yield of hybrid rice seed production has stagnated at 2.5 tons per hectare (Xie 2009). The low outcrossing rate of the maternal parent is the main factor limiting further increases in the F_1_ seed production yields in rice because it is a typically self-pollinated crop (Kato and Nimai 1987). The rice flowers receive pollen on their stigmas, and exserted stigmas extend past the floral organ known as a glume; rice stigmas remain receptive for approximately 4 days, and thus exserted stigmas have more opportunities to trap pollen from other rice genotypes, thereby improving cross-pollination rates(Long and Shu 2000; Tian et al. 2004). The stigma exsertion rate is a major factor that contributes to the efficient improvement of hybrid seed production.

Many studies have shown that stigma exsertion in rice is a complex quantitative trait governed by polygenetic inheritance. In the past two decades, with the development of genomics and molecular markers, a QTLs for stigma exsertion in rice have been mapped by using different segregating populations such as F_2_ populations (Xiong et al. 1999; Yue et al. 2009; Deng et al. 2010; Feng et al. 2010; Li et al. 2010; Deng et al. 2011; Chen et al. 2011; Li et al. 2017); recombinant inbred lines (Uga et al. 2003; Yamamoto et al. 2003; Shen et al. 2006; Yu et al. 2006; Yin et al. 2014; Li et al. 2014; Rahman et al. 2016); doubled haploid lines (Hittalmani et al. 2002; Li et al. 2003); backcrossing populations (Li et al. 2001; Miyata et al. 2007; Qiao et al. 2007; Qiao et al. 2008); and chromosome segment substitution lines (Liu et al. 2015; Rahman et al. 2017a; Rahman et al. 2017b). Genome-wide association studies (GWAS) have more recently been employed to identify loci associated with stigma exsertion (Yan et al. 2009; Huang et al. 2012; Dang et al. 2016; Zhou et al. 2017; Guo et al. 2017). The QTLs that affect stigma exsertion were distributed across all 12 rice chromosomes. Nevertheless, only a small handful of QTLs explained more than 10% of the phenotypic variation. And a small proportion of these QTLs have been fine mapped or cloned. The major QTL *qES3* was repeatedly identified and co-located with the grain size gene *GS3* (Yamamoto et al. 2003; Miyata et al. 2007). Takano-Kai et al. (2011) confirmed that *GS3* controls both stigma length and exsertion. They also demonstrated that a nonsense mutation in the second exon of *GS3* increased cell number in the stigma, resulting in elongation and exsertion of the stigma. Liu et al. (2015) fine mapped the QTL *qSTL3*, which is associated with stigma length, to a 19.8-kb region in the middle of the short arm of chromosome 3 and validated *LOC_Os03g14850* as a candidate gene associated with *qSTL3*. They also developed a gene-specific marker for improving the stigma length of the maternal parent, thereby increasing the outcrossing rate of the maternal parent in japonica hybrid seed production. Rahman et al. (2017a) dissected a major QTL (*qSE11*) and narrowed its location to a 350.7-kb region on rice chromosome 11. Despite so many QTLs having been identified, the genetic mechanism underlying stigma exsertion rate is poorly understood and requires further investigation.

In our previous study, the main effect QTL *qSER-7* was localized to the rice chromosome 7 region flanked by markers RM3859 and RM5436 using a F_2_ population derived from two indica CMS maintainers, Huhan 1B and II-32B. The positive effect of *qSER-7* was from the high stigma exsertion rate parent II-32B improved both single stigma exsertion and total stigma exsertion rates, explaining 8.12% and 8.15% phenotypic variation, respectively(Yue et al. 2009).

In the present study, we performed fine-linkage mapping of *qSER-7* by using a BC_4_F_2_ segregation population derived from Huhan 1B and II-32B. Ultimately, the location of *qSER-7* was narrowed down to a 28.4-kb region on rice chromosome 7 flanked by the RM3859 and Indel4373 markers. We also analyzed key candidate genes in that region that may be the target gene related to stigma exsertion. These results will be useful for facilitating the development of male sterile lines with high stigma exsertion rates, which would be of great value in hybrid rice seed production.

## Results

### Development of the near isogenic line for *qSER-7*

**Fig. 1:**
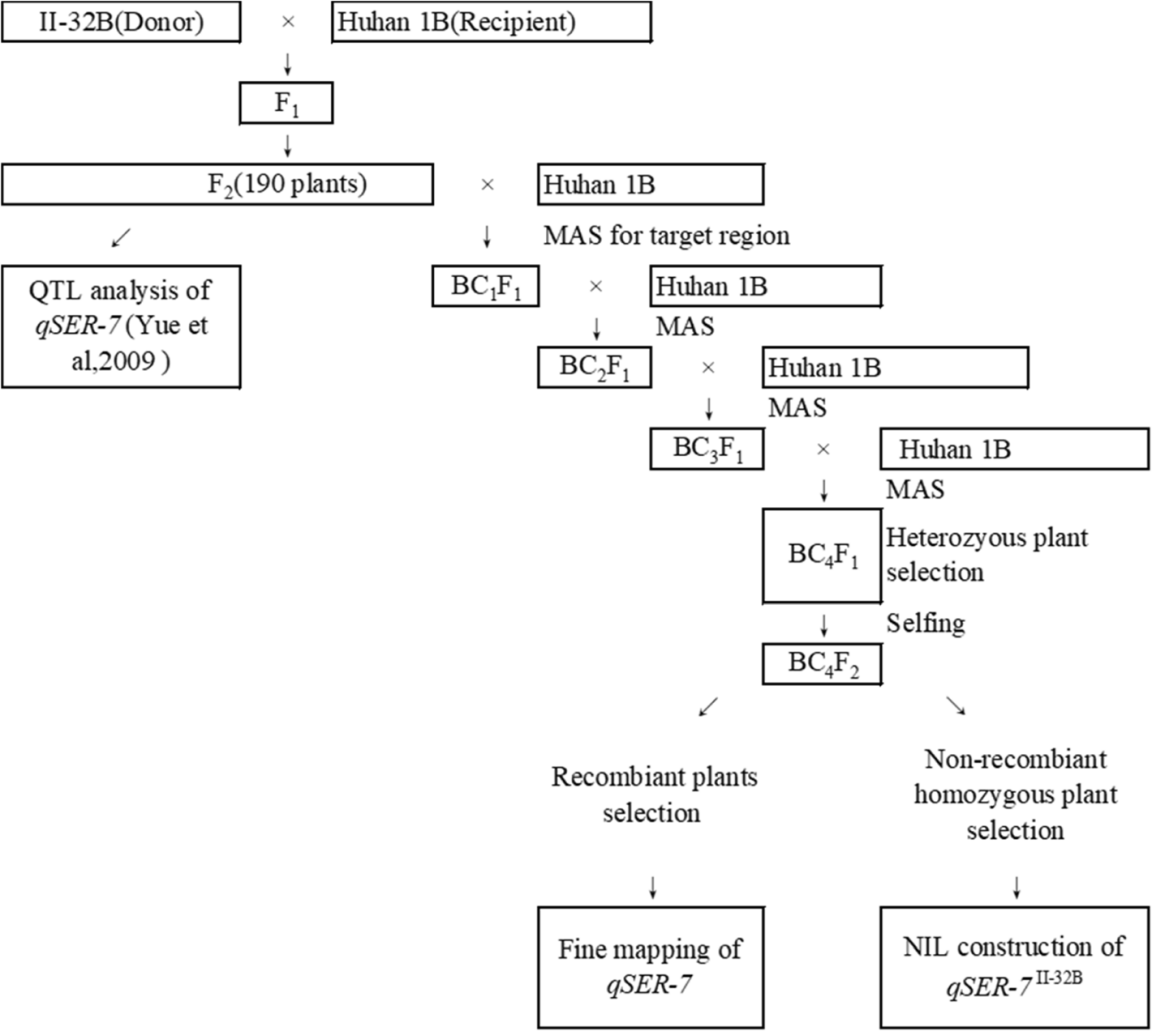
The scheme of plant population development for QTL analysis, NIL construction and QTL fine mapping

**Fig. 2:**
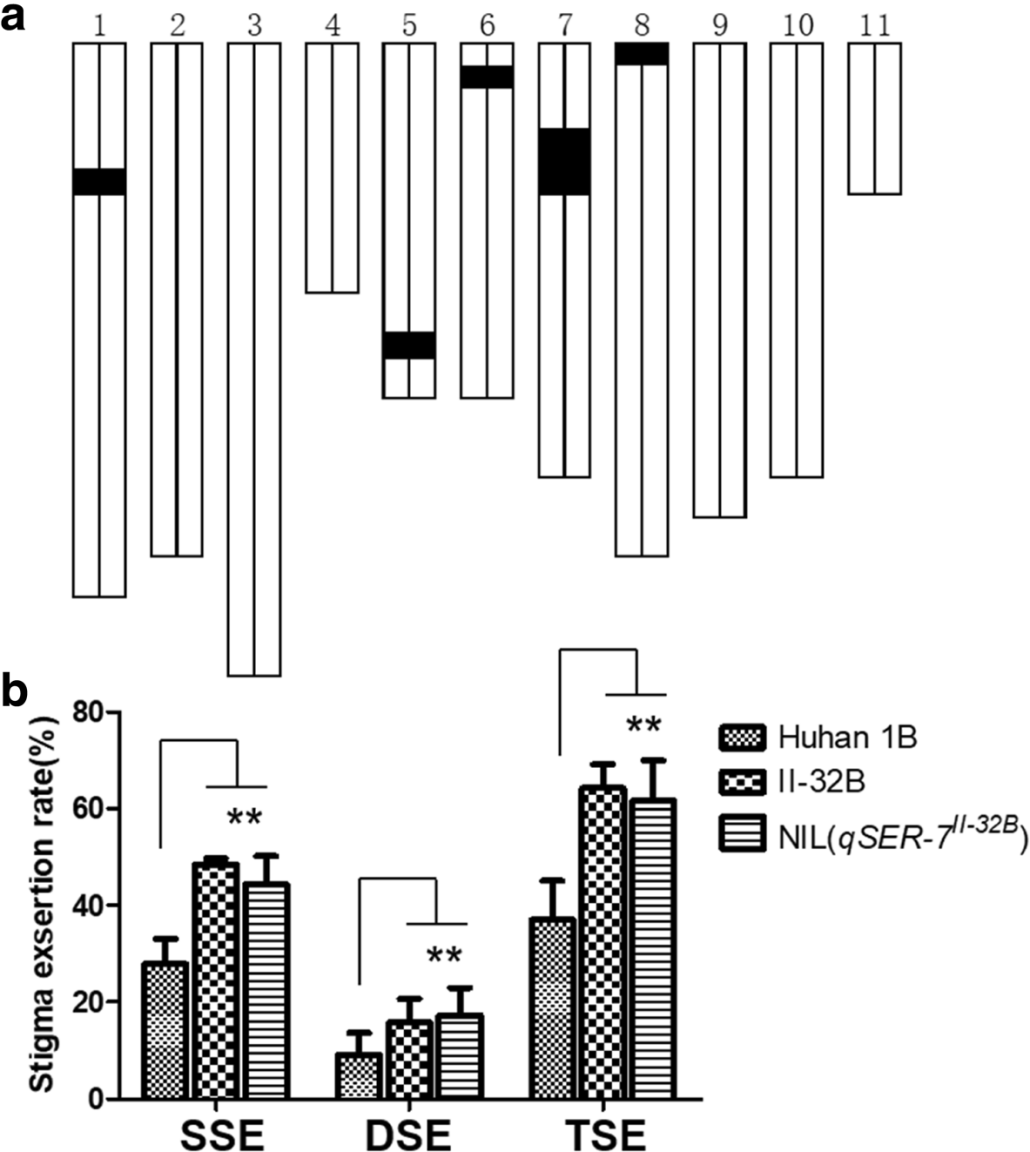
The development of NIL. **a** Graphical genotype of NIL (*qSER-7*^II-32B^). Black bar indicates the genome fragment from II-32B; the other parts were from Huhan 1B. **b** Stigma exertion rate for Huhan 1B, II-32B and NIL (*qSER-7*^II-32B^). SSE: single stigma exsertion rate, DSE: dual stigma exsertion rate, TSE: total stigma exsertion rate. The data represent the mean ± SD (*n* = 10), **P ≤ 0.01

Based on previous research, *qSER-7* was mapped to between markers RM3859 and RM5436 (Yue et al. 2009). Substitution mapping was used to isolate *qSER-7* and one line from the F_2_ population was selected for four rounds of backcrossing with Huhan 1B (Fig. 1). The simple sequence repeat (SSR) markers RM3859 and RM5436 were used in marker-assisted selection for segregating the progenies carrying the II-32B *qSER-7* allele during each backcross generation. After continuous backcrossing for four generations and selfing, the genetic background became relatively similar to that of the recurrent parent Huhan 1B except for the substituted target segments, for which the BC_4_F_2_ plants were scanned with a set of 102 SSR markers that were uniformly distributed across a previous linkage map (Additional file 1: Table S1). The individual plant exhibiting the maximum recurrent parent genome recovery (94.11%) was selected, i.e., NIL (*qSER-7*^II-32B^), which carried a homozygous introgression from II-32B across the entire *qSER-7* region in the Huhan1B genetic background (Fig. 2).

### Phenotypic and genetic analysis

**Fig. 3:**
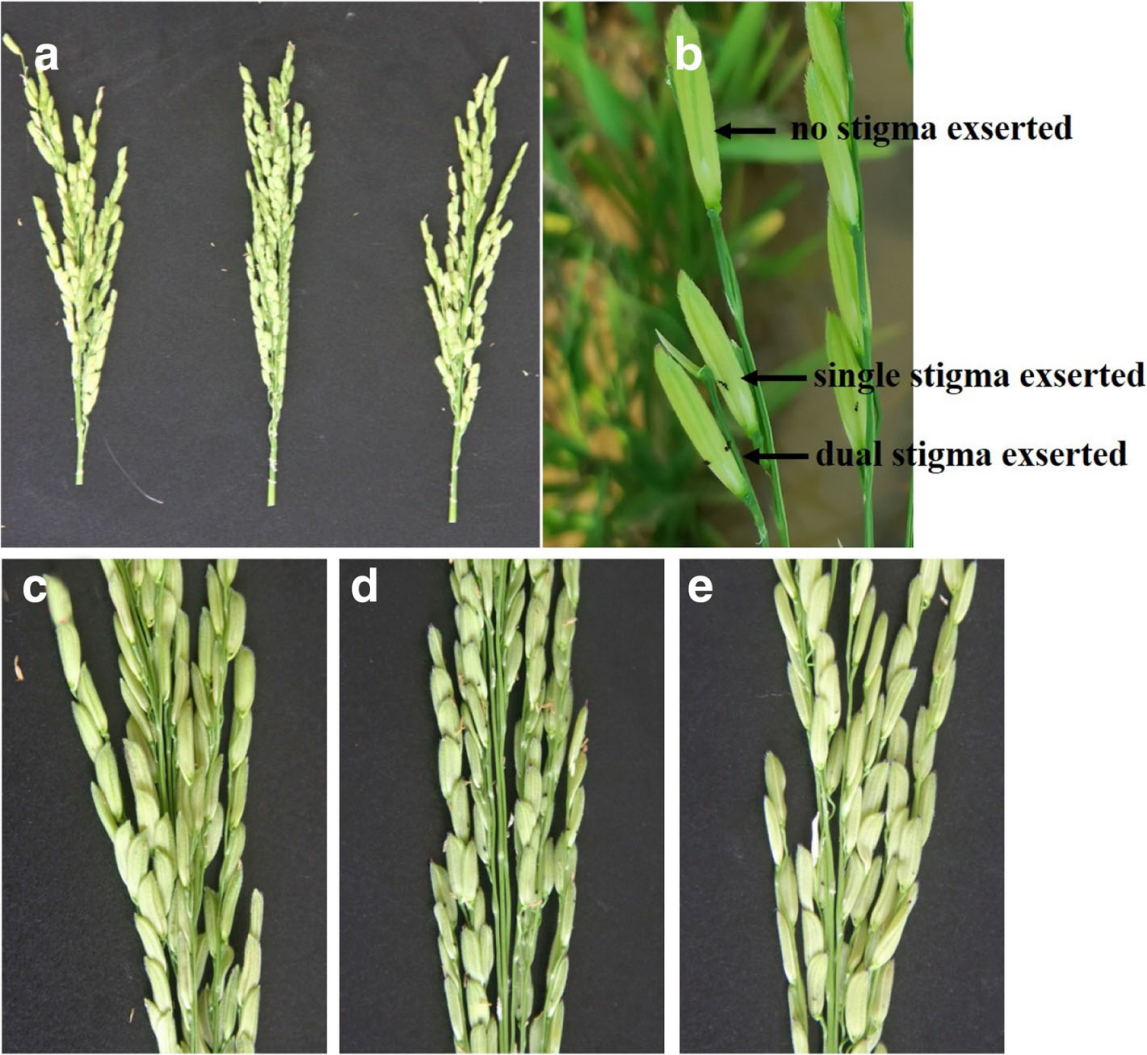
The phenotype of the stigma exsertion of the parents Huhan 1B, II-32B, and NIL (*qSER-7*^II-32B^). **a**The panicles of the parental lines shown from the left to the right are Huhan 1B, II-32B, and NIL (*qSER-7*^II-32B^). **b** Examples of single, dual, and no stigma exsertion in a spikelet, **c** Huhan 1B (stigmas are brown). **d** II-32B (stigmas are black). **e** NIL (*qSER-7*^II-32B^)

The stigma exsertion rate of the recurrent parent Huhan 1B was 27.93% (single stigma exsertion rate, SSE), 9.08% (dual stigma exsertion rate, DSE), and 37.01% (total stigma exsertion rate, TSE), whereas the NIL (*qSER-7*^II-32B^) phenotypic traits were higher (43.84%, 17.26%, and 61.10%, respectively). Thus, the NIL (*qSER-7*^II-32B^) had increased exsertion rates of 15.91%, 8.25% and 24.09% (SSE, DSE, and TSE, respectively), compared to the recurrent parent Huhan 1B (Table 1, Fig. 3). This result indicates that *qSER-7* is responsible for the high stigma exsertion rate in NIL (*qSER-7*^II-32B^).

**Table 1:** The stigma exsertion rates of Huhan 1B, II-32B and NIL (*qSER-7*^II-32B^)

Traits	Huhan 1B	II-32B	NIL (*qSER-7*^II-**32**B^)
Single stigma exsertion rate (%)	27.93 ± 5.00	48.50 ± 1.19	43.84 ± 7.49
Dual stigma exsertion rate (%)	9.08 ± 4.38	15.73 ± 4.74	17.26 ± 5.39
Total stigma exsertion rate (%)	37.01 ± 7.80	64.23 ± 4.77	61.10 ± 9.48

NIL (*qSER-7*^II-32B^), is a near isogenic line carrying the homozygous *qSER-7* region from II-32B on Huhan 1B genetic background. Trait values are shown as mean ± standard deviation values

Among the random selection of 120 individuals in the BC_4_F_2_ population, the marker RM3859 was used to validate the effect of *qSER-7*. The result of a Chi-squared test showed that the three genotypes were distributed according to 1:2:1 Mendelian ratio (χ^2^ = 1.38 < χ^2^_0.05,2_ = 5.99). The total stigma exsertion rates of II-32B homozygotes and heterozygotes were significantly higher than that of Huhan1B homozygotes, indicating that the effect of *qSER-7* is likely controlled by one genetic locus (Table 2).

**Table 2:** Marker segregation and total stigma exsertion rate of the three genotypic groups in the BC_4_F_2_ population

Marker	Numbers of plants in the three genotypic groups			*χ*^*2*^(1:2:1)	Phenotypic of the three genotypic groups		
	Huhan1B homozygote	Heterozygote	II-32B homozygote		Huhan1B homozygote	Heterozygote	II-32B homozygote
RM3859	25	61	34	1.38	39.16 ± 7.14^a^	57.36 ± 9.02^b^	62.17 ± 11.42^bc^

The superscript letters indicate statistically significant differences (*p* ≤ *0.01*) between the mean values within each row (Student’s t-test). Phenotypic values are shown as mean ± standard deviation values

### Homozygous recombinant plant selection and fine mapping of *qSER-7*

**Fig. 4:**
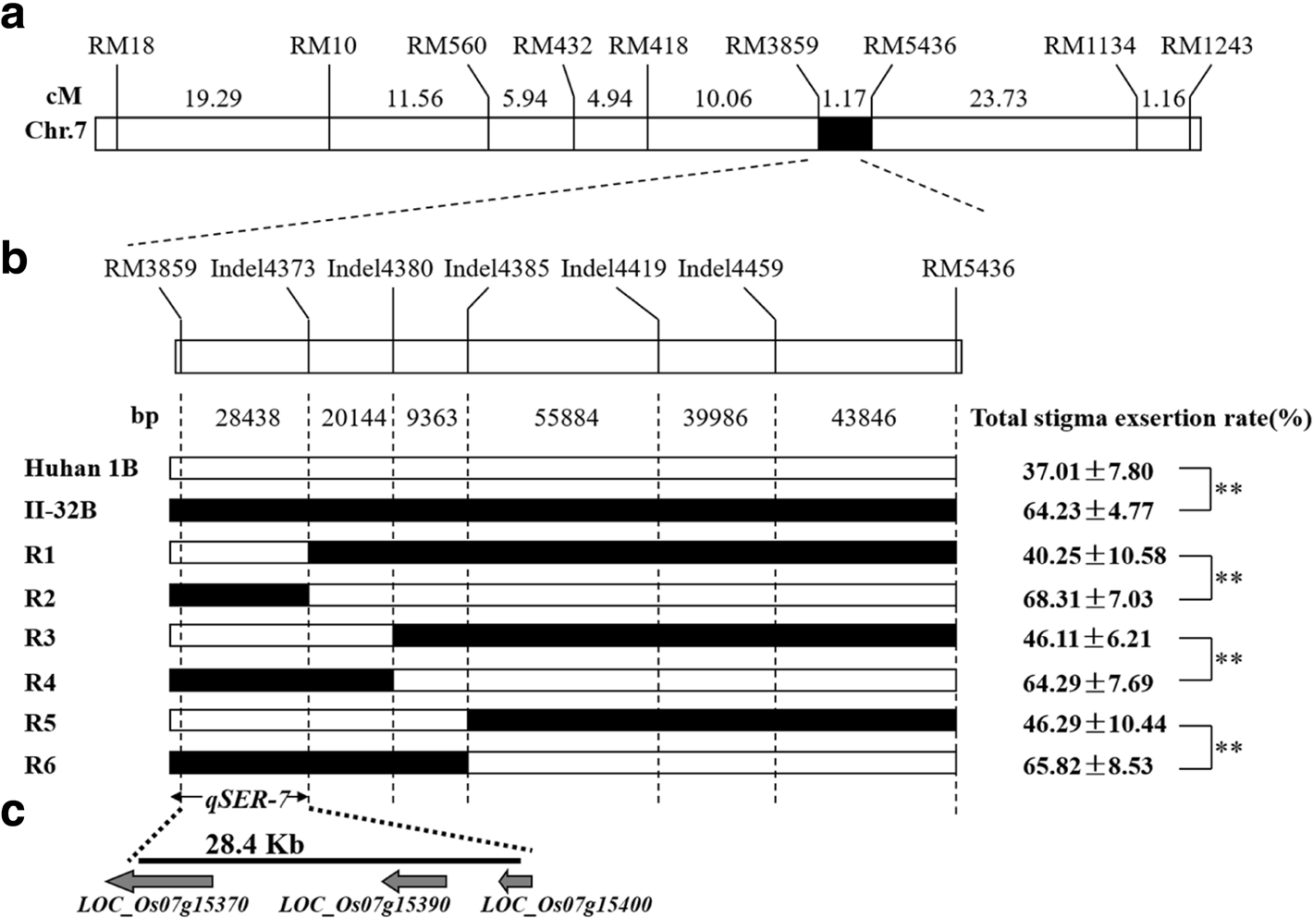
Fine mapping of *qSER-7.* **a** The genetic linkage map of *qSER-7* region on chromosome 7. The number between markers indicate genetic distance in centimorgans. **b** Fine mapping of the *qSER-7* locus with six homozygous recombinants. Genotypes and phenotypes of homozygous recombinant lines(R1-R6) were performed from BC_4_F_4_ population. The *qSER-7* was narrowed down to a 28.4-kb interval between the markers RM3859 and Indel4374. Black and white bars indicate homozygous for II-32B and Huhan 1B, respectively. The number between markers indicate physical distance. The data represent the mean ± SD (n = 10), ** P ≤ 0.01, t-test. **c** Candidate region of the *qSER-7* locus and the annotated genes in the rice genome annotation database (http:// rice.plantbiology.msu.edu/, MSU- version_7.0)

The SSR marker RM3859 on one side of the *qSER-7* target region and RM5436 on the other side were used to identify recombination break points in segregating progenies. To narrow down the location of *qSER-7*, we developed six additional InDel markers (Additional file 2: Table S2). A total of 3192 BC_4_F_2_ progenies were cultivated in order to screen for recombinants, and 18 heterozygous individuals were selected and selfed to generate individuals with homozygous genotypes. Six different BC_4_F_3:4_ homozygous recombinant lines (R1-R6) in the QTL region were analyzed for fine mapping. The phenotypic performance of the stigma exsertion rates varied from 40.25% to 68.31% (Fig. 4b) in the homozygous recombinant lines. The total stigma exsertion rates of R1, R3, and R5 were similar to that of the ‘Huhan 1B’ recurrent parent; however, R2, R4, and R6 had significantly higher stigma exsertion rates similar to that of II-32B donor parent. Based on the genetic and phenotypic analysis, the location of *qSER-7* was finally narrowed to a 28.4-kb region between the RM3859 and Indel4373 markers (Fig. 4b).

### Candidate gene analysis for *qSER-7*

**Fig. 5:**
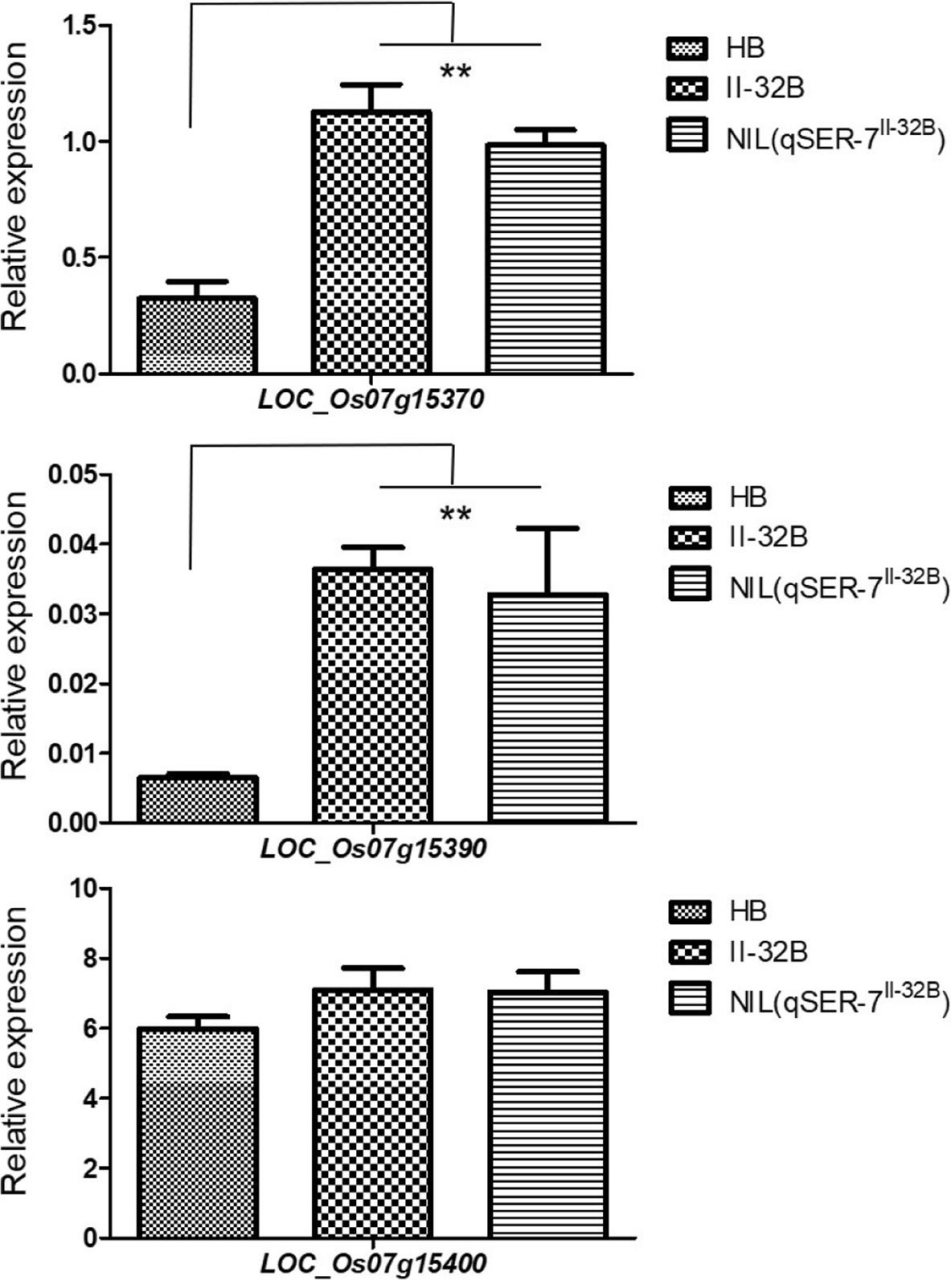
The RT-PCR analysis of predicted genes in Huhan 1B, II-32B, and NIL (*qSER-7*^II-32B^) RNA was isolated from frozen young panicles (5 mm to 100 mm). ** P ≤ 0.01, t-test. Values are the mean ± SD with three biological replicates.

According to the rice genome annotation database (http:// rice.plantbiology.msu.edu/, MSU- version_7.0), three genes were predicted in the mapping region: *LOC_Os07g15370*, *LOC_Os07g15390* and *LOC_Os07g15400* (Fig. 4c). *LOC_Os07g15370* (*OsNRAMP5*) was reported to be a Mn and Cd transporter involved in the root uptake of these metals from the medium (Ishikawa et al. 2012; Yang et al. 2014); *LOC_Os07g15390* and *LOC_Os07g15400* putatively encode a retrotransposed protein and expressed protein, respectively. We performed a quantitative reverse transcription-PCR (RT-PCR) analysis to determine the candidate genes, and the analysis revealed that the expression level of *LOC_Os07g15370* and *LOC_Os07g15390* were significantly higher in II-32B and NIL (*qSER-7*^II-32B^) compared with Huhan 1B, however, no significant difference was observed in *LOC_Os07g15400* (Fig. 5). Therefore, *LOC_Os07g15370* and *LOC_Os07g15390* were likely to be the candidate genes for *qSER-7*.

Sequence comparison between II-32B and Huhan1B revealed no coding sequence polymorphisms differentiating these two genes. For *LOC_Os07g15370*, a single 1-bp deletion was identified in the promoter region between the parental lines. For *LOC_Os07g15390*, 16 single-base substitutions were found in the promoter sequence (Additional file 3: Table S3), suggesting that the effect at *qSER-7* might result from a difference in expression levels between II-32B and Huhan1B alleles.

**Fig. 6:**
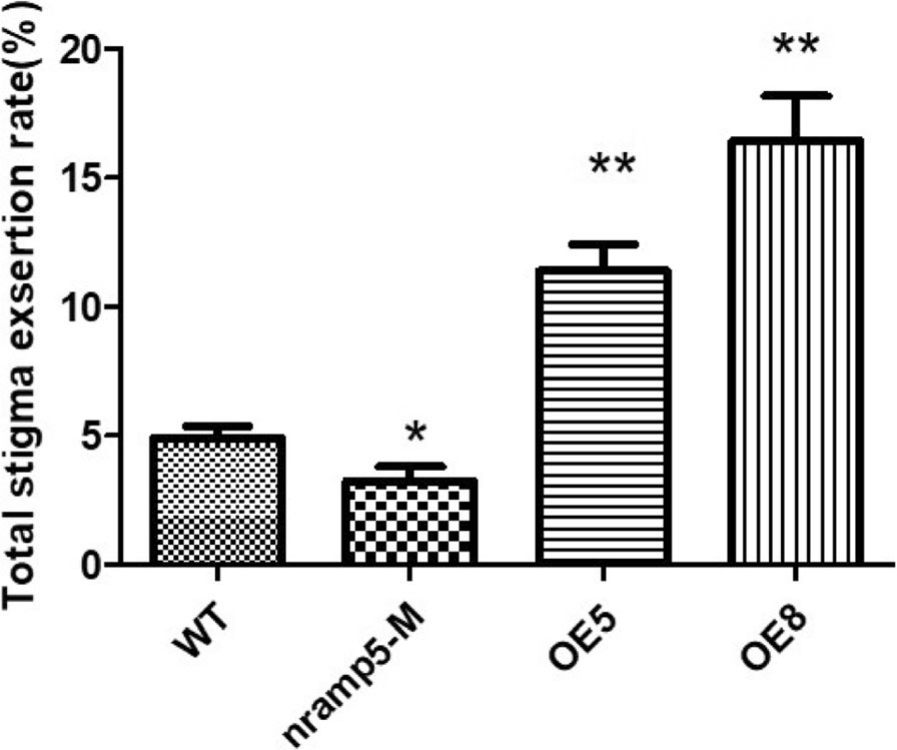
Stigma exertion rate assay of *osnramp5* mutant and *OsNRAMP5* overexpressing transgenic rice. WT: wild type, OE5 and OE8: overexpressing transgenic lines, *nramp5* M: *osnramp5* mutant. The data represent the mean ± SD (n = 10), *P ≤ 0.05, **P ≤ 0.01

A T-DNA insertion mutant *osnramp5*, *OsNRAMP5*-overexpressing transgenic rice plants and wild type (Zhonghua 11) plants were obtained from Yang et al. (2014) for preliminary phenotypic evaluation. Compared to wild type plants, the stigma exertion rates of *OsNRAMP5*-overexpressing plants (OE5 and OE8 lines) were significantly higher; in contrast, the stigma exertion rates of the *osnramp5* mutant was significantly lower than that of wild type plants (Fig. 6, Additional file 4: Figure S1).

## Discussion

Stigma exertion rate is a female parental trait that is essential for improving hybrid seed production in rice. Exerted stigmas are fragile and thus can be easily damaged by environmental conditions such as wind, water stress, and physical disruption during the flowering period (Yu et al. 2006; Yan et al. 2009). A number of SER QTLs have previously been detected and are distributed across all 12 chromosomes in rice. However, the results of these QTL locations are not exactly consistent across population types, mark densities and analytical methods used by different studies. Previous studies have demonstrated that stigma exertion rate is significantly positively correlated with stigma length (Kato and Nimai 1987; Miyata et al. 2007). Currently, *GS3* (Takano-Kai et al. 2011) and *qSTL3* (Liu et al. 2015) are the only two cloned genes, that have been shown to increase stigma exsertion by increasing stigma length. However, neither of these two genes were cloned based on studies in which stigma exertion rate was the target trait. Recently, a major QTL (*qSE11*) for stigma exertion rate was narrowed to a 350.7-kb region between the SE6–10 and SE10 markers on the long arm of rice chromosome 11 (Rahman et al. 2017a). One of the main reasons for slow progress in the fine-mapping of stigma exertion rate QTL is that stigma exertion is strongly influenced by environment. To improve the accuracy of the phenotype assay, ten main panicles of parental and homozygous recombinant lines were collected at 5–7 days after heading, when the lower spikelets of the panicle had flowered, and stored at − 20 °C. Observation and counting were performed by the same person. Thus, the data generated in the present study with this technique provides an accurate description of stigma exsertion rate in the genotypes studied.

Development of NILs is a productive strategy for QTL confirmation and evaluation of their genetic effect, and it provides useful materials for population development during QTL fine mapping (Ding et al. 2011). One of the parents used in this study, Huhan1B, is the maintainer line of Huhan 1A, which is the first indica CMS line of water-saving and drought resistance rice (WDR) (Luo 2010). The NIL of *qSER-7* regions were constructed using Huhan1B as the recurrent parent. The NIL had increased rates of exsertion frequency of 15.91%, 8.25% and 24.09% (SSE, DSE, and TSE, respectively), compared to the recurrent parent Huhan1B. Thus, an improved version of Huhan1A carrying a homozygous *qSER-7* region would shows a higher stigma exsertion rate and more hybrid seeds would be produced on a plant, and has therefore been widely used for WDR breeding.

A total of 10 QTLs for stigma exsertion rate were detected on chromosomes 3, 4, 7, and 9 in our previous study (Yue et al. 2009). The QTL flanked by RM3859 and RM5436 on chromosome 7, *qSER-7*, which had effects on single stigma exsertion and total stigma exsertion rate was dissected in this study. The *qSER-7* region coincides with QTLs reported by Yin et al. (2014). In our report, *qSER-7* was fine-mapped to a 28.4-kb region. The target region contains three predicted genes. *LOC_Os07g15370* and *LOC_Os07g15390* are likely to be the candidate genes according to the real time quantitative RT-PCR analysis. *LOC_Os07g15370* (*OsNRAMP5*) was reported to be a Mn and Cd transporter involved in the root uptake of these metals from the medium (Ishikawa et al. 2012; Sasaki et al. 2012). Yang et al. (2014) found that *OsNRAMP5* exhibited the strongest expression signal in young reproductive tissues, e.g., panicles and spikelets. Preliminary phenotypic evaluation showed that the stigma exertion rates of two *OsNRAMP5*-overexpressing plants (OE5 and OE8 lines) were significantly higher than that of wild type plants. Furthermore, more work, such as a transgenic complementary test, are needed to examine whether *LOC_Os07g15370* or *LOC_Os07g15390* is the most likely candidate underlying the effect of *qSER-7*. Dissection of the genetic mechanisms for stigma exertion rate, which will facilitate rice molecular breeding for high stigma exsertion rate, will continue to improve the efficiency of hybrid seed production.

## Conclusion

In this study using 3192 individuals from a BC_4_F_2_ segregation population, a new QTL (*qSER-7*) for stigma exsertion rate, was fine mapped to within a 28.4-kb physical interval on chromosome 7. Two candidate genes were finally selected based on differences in transcriptional expression. The cloning and examination of the genetic study of the effect of *qSER-7* will facilitate increasing the stigma exsertion rate of male sterile lines and the further improvement of hybrid rice seed production.

## Methods

### Population and cultivation

The detailed process of population development is illustrated in Fig. 1. In the previous study, the main effect QTL *qSER-7* was detected for single stigma exsertion and total stigma exsertion rates using a F_2_ population derived from a cross between Huhan 1B and II-32B (Yue et al. 2009). Huhan1B is the maintainer line for Huhan 1A, which is the first indica CMS line of water-saving and drought resistance rice (WDR) with a low stigma exsertion rate, whereas II-32B is the maintainer line of II-32A which has a good flowering habit and high stigma exsertion rate.

To obtain a relatively simple genetic background and to fine map the *qSER-7* locus, we constructed the NIL with respect to *qSER-7*. To this end, an F_2_ line with the II-32B genotype in the *qSER-7* region was selected to successively backcrossed with Huhan 1B for four generations. The SSR markers RM3859 and RM5436 were used for marker-assisted selection (MAS) of each generation among the segregating progenies. As a result, a BC_4_F_1_ line with the Huhan 1B genetic background, but exhibiting heterozygositys across the entire *qSER-7* region was constructed. After selfing, we acquired a BC_4_F_2_ line and used homozygous recombinants from the BC_4_F_4_ generation for fine mapping of *qSER-7*. Based on the genotypes of the II-32B alleles, one BC_4_F_2_ plant with homozygous II-32B regions surrounding the *qSER-7* allele with a single segment was chosen and named NIL (*qSER-7*^II-32B^). A set of 102 simple sequence repeat (SSR) markers that were uniformly distributed on a previous linkage map (Yue et al. 2009) were used to screen the genetic background (Additional file 1: Table S1).

The BC_4_F_1_ population was planted at Lingshui, Hainan Island, China, in winter 2015; the BC_4_F_2_ population was planted at Shanghai, China, in summer 2016. The BC_4_F_3_ and BC_4_F_4_ populations were planted at Hainan in winter 2016 and Shanghai in summer 2017, respectively. Recombinant plants, NIL (*qSER-7*^II-32B^) and their parents were established in five rows with seven plants per rows. Spacing was maintained at 30 cm between rows and 20 cm between plants. Standard crop management practices were followed.

Seeds of T-DNA insertion mutant, wild type (Zhonghua 11), and transgenic plants were obtained from Huazhong Agricultural University, Wuhan City, Hubei Province, China in 2016(Yang et al. 2014).

### Phenotypic evaluation

The stigma exsertion rate was subdivided into three traits, single stigma exsertion rate (SSE), dual stigma exsertion rate (DSE) and total stigma exsertion rate (TSE). At 5–7 days after all spikelets had flowered, ten main panicles from parents and homozygous recombinant lines were collected for the examination of stigma exsertion rates. SSE, DSE, and TSE were calculated as the percentage of the numbers of spikelets with single stigma exsertion, dual stigma exsertion, and either single or dual stigma exsertion in the total number of spikelets, respectively.

### DNA extraction and development of molecular markers

Total DNA was extracted from fresh leaves using the CTAB method (Murray and Thompson 1980). PCR was performed in 20-μL reaction volumes containing 1.5 μL of 20.0 ng/μL template DNA, 10 μL of Taq PCR Mastermix (TIANGEN, Beijing, China), 2 μL of 10 μmol/μL primer pairs, and 6.5 μL of ddH_2_O. The thermal cycling consisted of an initial denaturation at 95 °C for 5 min, followed by 35 cycles of denaturation at 95 °C for 30 s, annealing at 55 °C for 30 s and extension at 72 °C for 45 s, with a final extension at 72 °C for 5 min. The PCR products were separated on 6% non-denatured polyacrylamide gels and detected by silver staining (Creste et al. 2001). Insertion/deletion (InDel) markers (Additional file 2: Table S2) were developed from the target region to determine recombination sites and the genotype of recombinant progenies based on the published rice DNA polymorphism database (Shen et al. 2004). The sequences of markers were designed using Primer Premier 5.0 (PREMIER Biosoft, Palo Alto, CA, USA).

### RNA extraction and real-time PCR analysis

Total RNA was isolated from young panicles (Stage In7 to Stage In8, with a panicle lengths of 5 to 100 mm) of Huhan 1B and II-32B at the pre-heading stage using TRNzol-A+ Total RNA Reagent (TIANGEN, Beijing, China). cDNA was obtained via reverse transcription of total RNA using the PrimeScript RT reagent Kit (Takara Biotechnology, Dalian, China) and following the manufacturer’s instructions. Real-time PCR was conducted using Hard-Shell 96-Well PCR Plates (BIO-RAD, Hercules, CA, USA), utilizing the CFX96TM Real-Time System (BIO-RAD). The utilized reaction system contained 10 μL of 2 × SYBR Premix Ex TaqTM (Takara Biotechnology, Dalian, China), 20 ng of cDNA, and 0.1 μM of gene-specific primers (Additional file 2: Table S2) in a final volume of 20 μL. The thermal cycling conditions used were 95 °C for 30 s, followed by 40 cycles at 95 °C for 5 s, and 60 °C for 31 s, followed by a final extension stage. The housekeeping gene Actin2 was used as a reference gene for calculating the relative expression levels of each gene.

#### Sequence and statistical analysis

Gene-specific PCR primers were designed to amplify the promoters and coding regions of *qSER-7* candidate genes in the two parents (Additional file 2: Table S2). The reaction mixture (50 μL) for the sequence analysis consisted of 2 μL of template DNA/cDNA, 5 μL of KOD-PCR Buffer, 5 μL of 2 mM dNTPs, 1 μL each of 10 mM forward and reverse primers, 3 μL of 2.5 mM MgSO_4_, 0.5 U of KOD enzyme, and 32 μL of ddH_2_O. The thermal cycling program included an initial denaturation at 95 °C for 5 min, followed by 35 cycles of denaturation at 95 °C for 30 s, annealing at 50 °C for 30 s and extension at 68 °C for 1 min, with a final extension at 68 °C for 5 min. The PCR products were then sub-cloned into the pEASY-Blunt cloning vector (TransGen Biotech, Beijing, China) according to the manufacturer’s protocol. Positive clones were sequenced by the BioSune Company (Shanghai, China). The sequence results were analyzed using DNASTAR software (DNASTAR Inc., Madison, WI, USA). All statistical analyses were performed using Excel (Microsoft Corp., Redmond, WA, USA) and SPSS 17.0 (SPSS Corp., Chicago, IL, USA).

## Additional files

Additional file 1: **Table S1.** SSR markers selected to screen the genetic background. (DOCX 16 kb)

Additional file 2: **Table S2.** Primer sequences designed in this study. (DOCX 16 kb)

Additional file 3: **Table S3.** Comparison of promoter sequences of two candidate genes between two parents. (DOCX 15 kb)

Additional file 4: **Figure S1.** Relative expression levels of *LOC_Os07g15370* in transgenic rice lines. (DOCX 55 kb)

## Data Availability

All relevant data are provided as tables within the paper in the additional files.

## Abbreviations

CMS: Cytoplasmic male sterility
DSE: Dual stigma exsertion rate;
GWAS: Genome-wide association study
InDel: Insertion/deletion
MAS: Marker-assisted selection
NIL: Near isogenic line
QTL: Quantitative trait locus
SER: Stigma exsertion rate
SSE: Single stigma exsertion rate
SSR: Simple sequence repeat
TSE: Total stigma exsertion rate
WDR: Water-saving and drought resistance rice
