# Supplementary material for: Fine mapping a quantitative trait locus, qSER-7, that controls stigma exsertion rate in rice (Oryza sativa L.)
Source: Rice (N Y). 2019 Jul 9;12:46. doi: 10.1186/s12284-019-0304-z (PMC6616572; doi:10.1186/s12284-019-0304-z)
Supplement: Supplementary file 1 — Table S1. SSR markers selected to screen the genetic background. (DOCX 16 kb) [file 12284_2019_304_MOESM1_ESM.docx]

| Table S1 SSR markers selected to screen the genetic background | | | |  |  |  |  |
| --- | --- | --- | --- | --- | --- | --- | --- |
| **Chromosome** | **SSR markers** |  |  |  |  |  |  |
| Chr.1 | RM495, RM562, RM488, RM128, RM302, RM212, RM102, RM5422, RM1160, RM472 | | | | | |  |
| Chr.2 | RM110, RM279, RM7082, RM6911, RM106, RM208 | | |  |  |  |  |
| Chr.3 | RM60, RM6959, RM251, RM6832, RM16, RM168, RM520, RM468 , RM422, RM514, R3M37 | | | | | |  |
| Chr.4 | RM3843, RM6909, RM1113, RM127, RM280, RM567, RM559 | | | |  |  |  |
| Chr.5 | RM39, RM7081, RM7446, RM6545, RM6972, RM3790, RM421 | | | |  |  |  |
| Chr.6 | RM508, RM540, RM197, RM588, RM587, RM510, RM217, RM584, RM225, RM314, RM111, RM276 | | | | | | |
| Chr.7 | RM1243, RM1134, RM418, RM432, RM5436, RM3859, RM560, RM10, RM18 | | | | |  |  |
| Chr.8 | RM407, RM1376, RM6999, RM7080, RM6208, RM7027, RM1309, RM339, RM223, RM5493, RM6765, RM6948, RM3840, RM447, RM264 | | | | | | |
| Chr.9 | RM444, RM105, RM242, RM278, RM3787, RM201, RM328, RM160, RM107, RM6971, RM6816, RM7306, RM2144, RM6294, RM1026, RM6797, R9M10 | | | | | | |
| Chr.10 | RM5348, RM228, RM590, RM591, R10M30 | | |  |  |  |  |
| Chr.11 | RM202, RM5349, RM21 | |  |  |  |  |  |
| Underlined markers indicate that introduced segments in the NIL from II-32B | | | | |  |  |  |
