# Supplementary material for: Fine mapping a quantitative trait locus, qSER-7, that controls stigma exsertion rate in rice (Oryza sativa L.)
Source: Rice (N Y). 2019 Jul 9;12:46. doi: 10.1186/s12284-019-0304-z (PMC6616572; doi:10.1186/s12284-019-0304-z)
Supplement: Supplementary file 2 — Table S2. Primer sequences designed in this study. (DOCX 16 kb) [file 12284_2019_304_MOESM2_ESM.docx]

| Table S2 Primer sequences designed in this study | | |  |  |
| --- | --- | --- | --- | --- |
| **Marker** | **Marker Type/Gene ID** | **Forward primer (5’-3’)** | **Reverse primer (5’-3’)** | **Purpose** |
| RM3859 | SSR | TTGCAGATCGGTTTCCACTG | GGTCCTGGATTCATGGTGTC | Linkage analysis |
| Indel4373 | InDel | AGTCTTTGATGGAAAGCTCC | TATTCCCAAAATTTTCATGG | Fine mapping |
| Indel4380 | InDel | AGGATCAGCTGAGAGACAAG | ACGTCGCAGAAGATGATG | Fine mapping |
| Indel4385 | InDel | TCAAGGACTAATTTGCAGAA | AAGATGGCACTTTACTCAAT | Fine mapping |
| Indel4419 | InDel | TCGCATTTGTGAATTAGTTG | AGCATAGCTCTGAAAACAGG | Fine mapping |
| Indel4459 | InDel | AAAATGTTTTACCACGCAAT | ATAGGAAACAATGAACACGG | Fine mapping |
| Indel4477 | InDel | TTCTTTAAGCAATCAAAGGG | CGTCCTACATGTGCAAAATA | Fine mapping |
| RM5436 | SSR | CAAAGGGGGTGTCCTCTATG | GTTGCTCGTCCTACATGTGC | Linkage analysis |
| cg1-CDS | *LOC_Os07g15370* | ATGGAGATTGAGAGAGAGAGCAGT | CTACCTTGGGAGCGGGATGT | Gene cloning |
| cg1-PRO | *LOC_Os07g15370* | CGCAACTCCCACAACTACTG | GCTTCCTCTCTTAGCTTCTTCA | Promoter amplify |
| cg2-CDS | *LOC_Os07g15390* | ATGCCGCCGCCGCTCGT | TTAAGCCATTAGTAGACAGATGTTT | Gene cloning |
| cg2-PRO | *LOC_Os07g15390* | ACCCACAACTAACGAACAAGC | CATATTCCTCCCTACAGCAGC | Promoter amplify |
| cg1 | *LOC_Os07g15370* | GGAATCAAGGACGGGTGC | CCAGATGCCAACAGTGCC | Real time RT-PCR |
| cg2 | *LOC_Os07g15390* | AGAGAATGGGAGGAGATGGG | CAAAAGGCTCACAGAGGTCAG | Real time RT-PCR |
| cg3 | *LOC_Os07g15400* | TGCGTCTTCTTCCTCTGTCAC | CAGTGTTCTTGGTGGATGTGG | Real time RT-PCR |
| Actin2 | *LOC_Os10g36650* | TTATGGTTGGGATGGGACA | AGCACGGCTTGAATAGCG | Real time RT-PCR |
