# Supplementary material for: Fine mapping a quantitative trait locus, qSER-7, that controls stigma exsertion rate in rice (Oryza sativa L.)
Source: Rice (N Y). 2019 Jul 9;12:46. doi: 10.1186/s12284-019-0304-z (PMC6616572; doi:10.1186/s12284-019-0304-z)
Supplement: Supplementary file 3 — Table S3. Comparison of promoter sequences of two candidate genes between two parents. (DOCX 15 kb) [file 12284_2019_304_MOESM3_ESM.docx]

| Table S3 Comparison of promoter sequences of two candidate genes between two parents | | | | | | | | | | | | | | | | | |
| --- | --- | --- | --- | --- | --- | --- | --- | --- | --- | --- | --- | --- | --- | --- | --- | --- | --- |
|  | LOC_Os07g15370 Promoter | LOC_Os07g15390 Promoter | | | | | | | | | | | | | | | |
| Positions of bases | –1,645 | –1,852 | –1,733 | –1,172 | –1,086 | –1,241 | –983 | –965 | –748 | –697 | –444 | –408 | –325 | –303 | –259 | –215 | –139 |
| Huhan 1B | – | A | A | A | A | T | G | A | G | A | G | C | T | T | T | G | A |
| II-32B | T | G | G | G | G | C | A | G | C | C | A | T | C | G | C | T | C |
