# Supplementary material for: Fine mapping a quantitative trait locus, qSER-7, that controls stigma exsertion rate in rice (Oryza sativa L.)
Source: Rice (N Y). 2019 Jul 9;12:46. doi: 10.1186/s12284-019-0304-z (PMC6616572; doi:10.1186/s12284-019-0304-z)
Supplement: Supplementary file 4 — Figure S1. Relative expression levels of LOC_Os07g15370 in transgenic rice lines. (DOCX 55 kb) [file 12284_2019_304_MOESM4_ESM.docx]

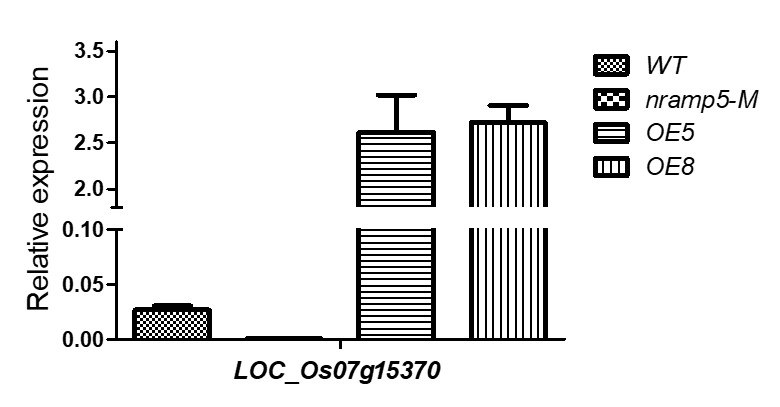


Fig.S1 Relative expression levels of *LOC_Os07g15370* in transgenic rice lines. WT: wild type, *nramp5* M: *osnramp5* mutant, OE5 and OE8: overexpressing transgenic lines. The gene expression level was analyzed by quantitative real-time PCR. The data represent the mean ± SD (n = 3)
